# Influence of Surfaces on Ion Transport and Stability in Antiperovskite Solid Electrolytes at the Atomic Scale

**DOI:** 10.1021/acsmaterialslett.4c01777

**Published:** 2024-10-10

**Authors:** Ana C. C. Dutra, James A. Quirk, Ying Zhou, James A. Dawson

**Affiliations:** † Chemistry−School of Natural and Environmental Sciences, Newcastle University, Newcastle upon Tyne NE1 7RU, U.K.; ‡ The Faraday Institution, Didcot OX11 0RA, U.K.

## Abstract

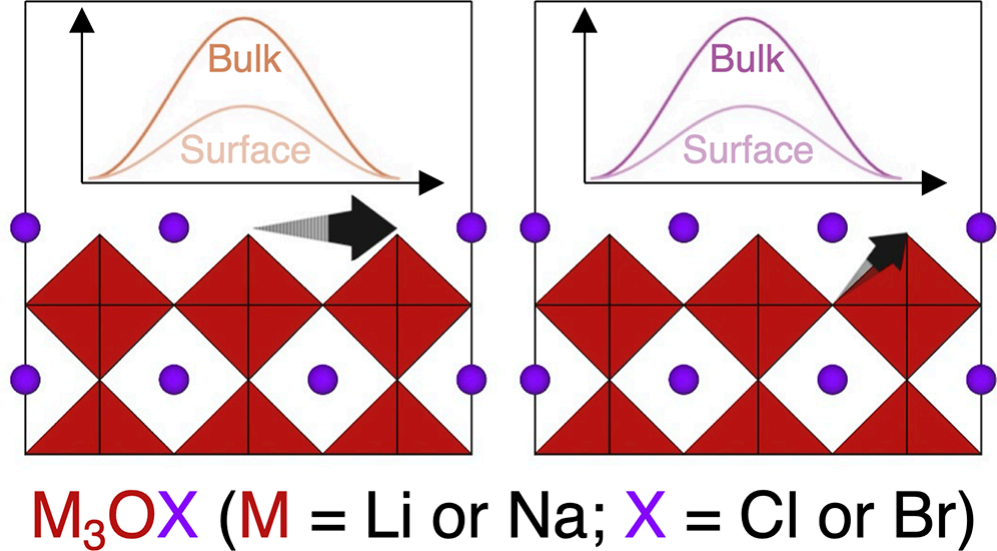

Antiperovskites are generating considerable interest as potential solid electrolyte materials for solid-state batteries because of their promising ionic conductivity, wide electrochemical windows, stability, chemical diversity and tunability, and low cost. Despite this, there is a surprising lack of a systematic study of antiperovskite surfaces and their influence on the performance of these materials in energy storage applications. This is rectified here by providing a comprehensive density functional theory investigation of the surfaces of M_3_OX (M = Li or Na; X = Cl or Br) antiperovskites. Specifically, we focus on the stability, electronic structure, defect chemistry, and ion transport properties of stable antiperovskite surfaces and how these contribute to the overall performance and suitability of these materials as solid electrolytes. The findings presented here provide critical insights for the design of antiperovskite surfaces that are both stable and promote ion transport in solid-state batteries.

Solid-state batteries are now widely considered as a next-generation technology with the potential to revolutionize energy storage. The core of solid-state battery technology lies in the substitution of flammable liquid electrolytes with solid counterparts, a replacement that leads to a plethora of proposed benefits, including improved energy density, voltage, cycle life, safety profiles and reduced costs.^1−3^ Nonetheless, several challenges still hinder the widespread use and commercialization of solid-state batteries, such as lithium dendrite growth, low ionic conductivity, electrochemical stability and interfacial resistance.^4−6^

Despite the significant progress made in the past decade in terms of discovering and assessing different solid electrolyte candidates, the incorporation of these novel materials into high-performance batteries remains challenging. This is primarily because of interfacial challenges, such as reduced conductivity, chemical incompatibilities, undesirable electrochemical reactions, degradation product formation and inferior mechanical performance.^2,7−9^ Therefore, new solid electrolytes that are only considered as promising based solely on their performance as a single isolated material without considering its interfaces may be revealed as being incompatible for solid-state battery applications. Given that the success of future solid-state battery implementations relies heavily on the engineering of more synergic interfaces, the understanding of such regions, their underlying mechanisms and the surfaces and materials that form them is vital for practical applications. However, despite recent progress,^10−12^ the current understanding regarding such topics at the atomistic scale is still limited compared to that of bulk materials because of the inherent difficulty in studying such interfaces, both computationally and experimentally.^13−19^

Among the many families of solid electrolytes, antiperovskites represent promising solid electrolyte candidates due to their excellent ensemble of features, including good ionic conductivity, stability against metal anodes, wide electrochemical windows, low-cost synthesis and a flexible and versatile structure.^20−23^ Considering these advantageous properties, several computational studies have investigated the interfacial properties of Li-based antiperovskites. While most of these studies have focused on the interfaces between antiperovskites and electrodes,^24−26^ some have considered the energetics and Li-ion transport properties of antiperovskite surfaces (i.e., with a vacuum).^27,28^ Nevertheless, as most studies focus solely on analyzing the archetypal Li_3_OCl antiperovskite, the understanding regarding the features and mechanisms pertinent to interfaces containing other Li-based antiperovskites (e.g., Li_3_OBr) is still limited. Additionally, there have not yet been any equivalent atomistic modeling studies of Na-based antiperovskite surfaces and there is therefore no critical consensus on the role of surfaces in determining the ion transport and stability of antiperovskite solid electrolytes. Furthermore, such understanding and the development of surface models are pivotal to building other important interfacial models, including electrode–electrolyte interfaces and grain boundaries, which are well-known to exhibit significant ion transport resistance.^10,17^

Based on the above, this study advances the understanding of M_3_OX (M = Li or Na; X = Cl or Br) antiperovskite solid electrolytes by exploring the stability, electronic structure, defect chemistry and ion transport properties of their surfaces using density functional theory (DFT) calculations. Our calculations show that the (100) surfaces with metal-halide terminations are the most thermodynamically stable surface type for all analyzed systems. Analysis of the electronic structure at the surfaces reveals that the Na-based systems could present lower electrochemical stabilities than their Li counterparts in solid-state battery configurations. We find that that Li and Na vacancies at the surface are the most energetically favorable defects for all the systems investigated. Additionally, our simulations predict that the cation (Li or Na) has a greater impact on defect formation at the surfaces of the investigated antiperovskites compared to the choice of halide (Cl or Br), with the formation of any defect at any site being most energetically favorable in systems containing Na instead of Li, regardless of the halide present. The calculated migration energy barriers illustrate the importance of accounting for the free lattice space between metal sites when designing new solid electrolytes, with our results suggesting the existence of a trade-off between choosing highly polarizable atoms and losing critical free space in the lattice for ionic migration. These findings provide critical insights for the design of solid electrolyte surfaces that are both stable and promote ion transport in solid-state batteries.

Prior to exploring the surface properties of M_3_OX (M = Li or Na; X = Cl or Br), it is necessary to determine the lowest energy (most stable) surface cuts and terminations for each analyzed system. The stability of six nonstoichiometric symmetric low-index surfaces for M_3_OX antiperovskites was examined by calculating their respective surface energies. Specifically, we considered two (100) surfaces with either a metal-halide or metal–oxygen termination, two (110) surfaces with either a metal or mixed metal–oxygen-halide termination and two (111) surfaces with either a metal-halide or oxygen termination.

The surface energies (*E*_surf_) were calculated from
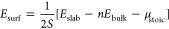
1where *E*_slab_ and *E*_bulk_ are the calculated energies of the slab and bulk unit cell, respectively, with *n* representing the number of bulk unit cells contained in the slab structure. The surface area is represented by *S* and *μ*_stoic_ is the total chemical potential of the metal oxide/halide, metal and/or oxygen atoms that must be removed to achieve a stoichiometric slab. For example, the (100) Li_3_OCl slab with ten atomic layers has a formula of Li_31_O_10_Cl_11_, which means that one lithium atom and one chlorine atom need to be removed to enable comparison with the equivalent stoichiometric system (Li_30_O_10_Cl_10_). The chemical potential of these removed species is approximated using the calculated energy of their respective compound and/or gas molecule, i.e., LiCl in this example. This procedure has previously been used to successfully calculate surface energies for nonstoichiometric Li_3_OCl^27^ and Li_3_OBr^28^ slabs. The optimized surface models and calculated surface energies for the antiperovskite systems are displayed in Figure 1a and b, respectively. No significant surface reconstruction was observed for any of the models.

**Figure 1:**
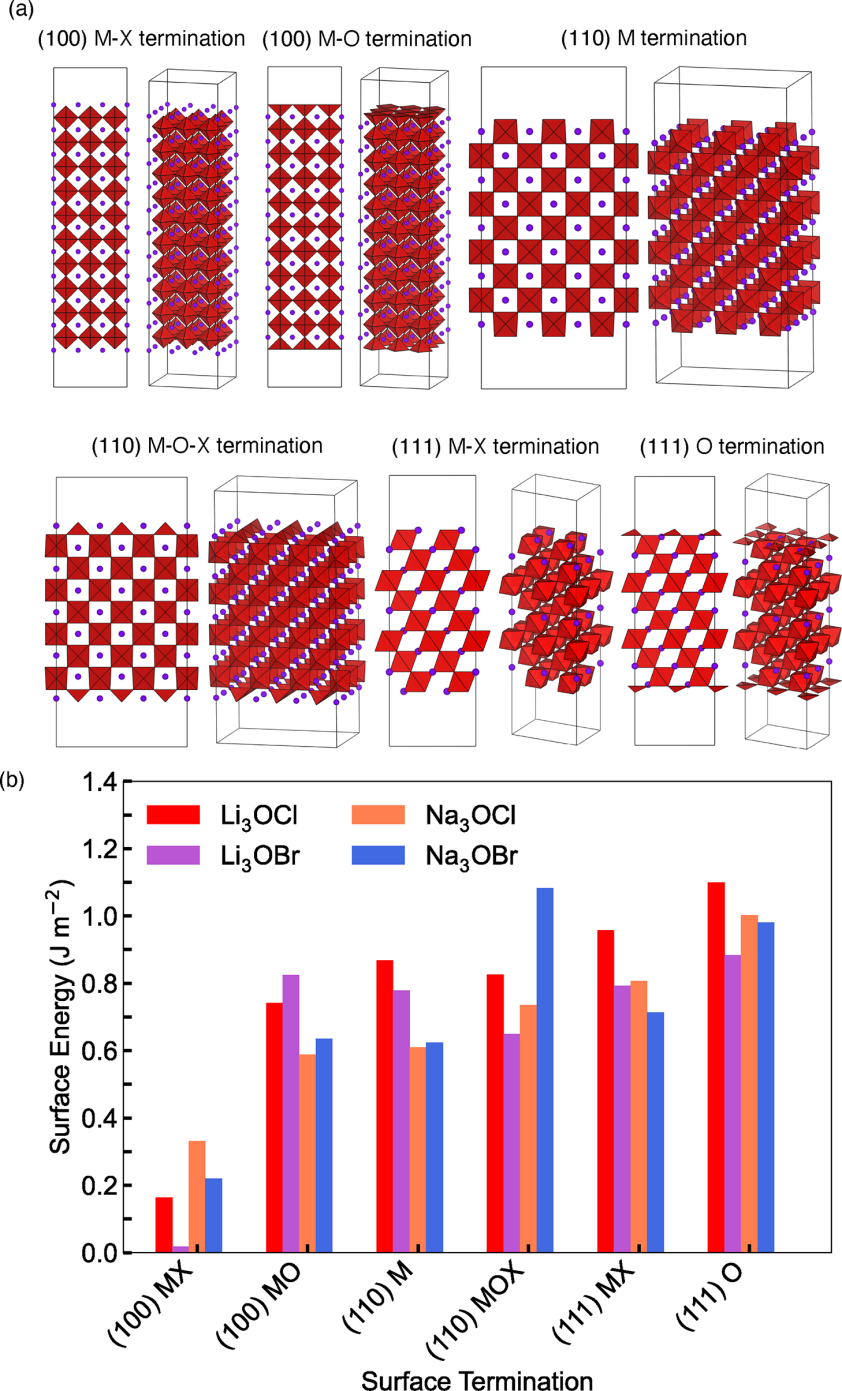
(a) Optimized surface models, where MO_6_ octahedra and halide atoms are red and purple, respectively, and (b) calculated surface energies for M_3_OX (M = Li or Na; X = Cl or Br).

The lowest surface energies were consistently found for the metal-halide-terminated (100) surface with values of 0.164, 0.018, 0.332, and 0.220 J m^–2^ for Li_3_OCl, Li_3_OBr, Na_3_OCl and Na_3_OBr, respectively. Given that a lower surface energy indicates its more energetically favorable formation, these results reveal that the (100) surface with metal-halide terminations is by far the most thermodynamically stable surface for all the analyzed systems. This preference for the metal-halide-terminated (100) surface agrees with previous findings for Li_3_OCl reported by Kim and Siegel^24^ and Wu et al.^27^ and for Li_3_OBr by Luo et al.^28^ The low energy for this surface can be explained through a simple electrostatic argument, in that only singly charged metal and halide ions are undercoordinated at the surface rather than doubly charged oxygen ions, as found for other solid electrolyte surfaces.^29^ Our calculated surface energies for the low-index surfaces of Li_3_OCl and Li_3_OBr are in excellent agreement with those calculated previously.^24,27,28^

In contrast to the Li-based materials, the surface energies for Na_3_OCl and Na_3_OBr have not been previously reported despite the significant amount of research attention these materials have received.^30−33^ As shown in Figure 1(b), the surface energies of the sodium-halide-terminated (100) surfaces are higher than for their Li counterparts but are still low compared to the other surfaces. The metal-bromide-terminated (100) surface is predicted to be more stable than the metal-chloride-terminated (100) surface for both the Li- and Na-based materials, possibly due to the increased softness and polarizability of the Br ions compared to the Cl ions,^34^ which can weaken the interaction between the Li/Na ions and surrounding anions.^35,36^ Nevertheless, there are several competing factors, including free volume, composition, electrostatics and polarizability, that determine the order of the stabilities of the different surfaces. This makes it difficult to explain the different trends that are observed for different surface terminations with absolute certainty. It is also important to consider the thermodynamic stability of the bulk phases in the context of surface formation. The convex hulls of these materials have been calculated several times in the literature and it is known that while Na_3_OCl and Na_3_OBr are considered stable with negative energies relative to the convex hull, Li_3_OCl and Li_3_OBr are metastable with slightly positive energies relative to the convex hull.^22,37,38^ The metastability of the Li-based antiperovskites may make it energetically easier to form the surfaces, hence resulting in the observed low surface energies for these materials. The metal-halide-terminated (100) surface was adopted for the electronic structure, intrinsic defect formation and ion transport investigations of all four antiperovskites, as discussed below.

An ideal solid electrolyte will display high electrochemical stability, negligible electronic conduction and no dendrite formation over cycling and these properties are inherently linked to the electronic structure of the solid electrolyte. To investigate the impact of surfaces on the electronic properties of antiperovskite solid electrolytes, the density of states (DOS) was calculated using the HSE06 hybrid functional for both the bulk materials and their metal-halide-terminated (100) surfaces, as shown in Figures S1 and 2(a), respectively. The valence band maximum (VBM) and conduction band minimum (CBM) are dominated by the O 2p and Li 2p states, respectively, in the bulk and surface models.

**Figure 2:**
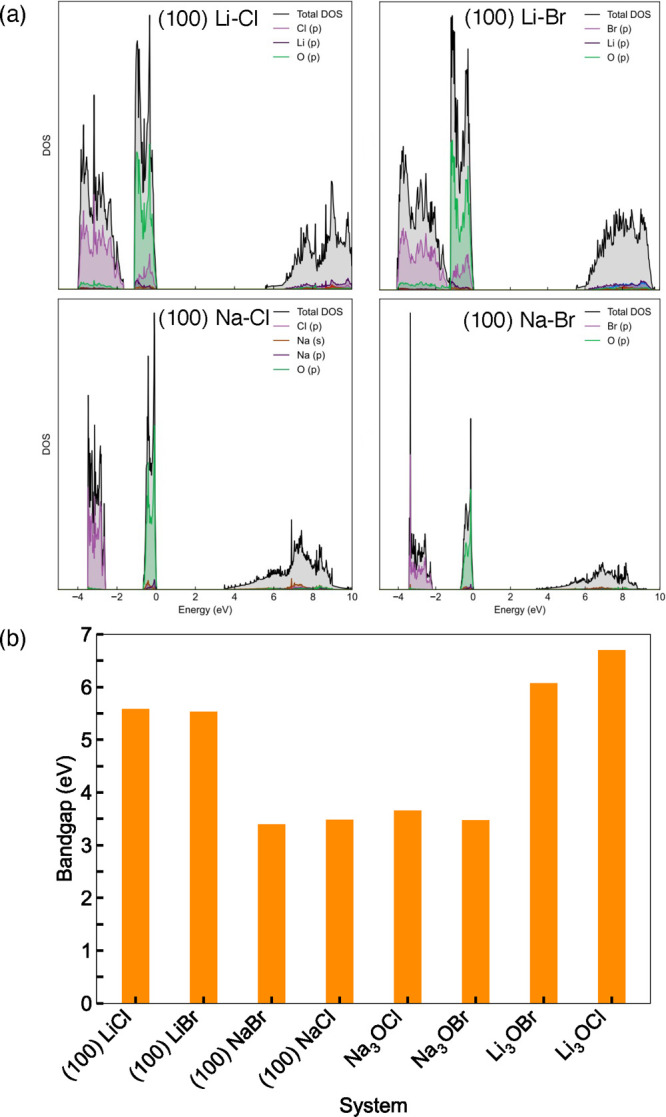
(a) DOS of metal-halide-terminated (100) surfaces with the VBM set to zero and (b) calculated bandgaps for M_3_OX (M = Li or Na; X = Cl or Br).

The calculated bandgaps from the DOS are given in Figure 2(b). Our calculated bandgaps for the bulk materials (Li_3_OCl (6.7 eV), Li_3_OBr (6.1 eV), Na_3_OCl (3.7 eV) and Na_3_OBr (3.5 eV)) are in good agreement with those previously reported in the literature,^39−41^ as are the bandgaps of 5.6 and 5.5 eV for the metal-halide-terminated (100) surfaces of Li_3_OCl and Li_3_OBr, respectively.^24,25^ Our results reveal that the bandgaps for the Na-based systems are significantly lower than for their Li counterparts. In contrast, the effect of changing the halide on the bandgap is not as significant, with only a small decrease in bandgap observed from Cl to Br. By comparing the bandgap results for the bulk and surface models in Figure 2(b), the surfaces clearly present slightly smaller values because of the introduction of additional states above the VBM and below the CBM. Previous computational work has revealed that such narrowed band gaps at interfaces can be indicative of electron and hole trapping that is highly localized at the vicinity of surfaces due to the presence of undercoordinated atoms.^10^ A large bandgap (and therefore negligible electronic conductivity), as observed for all the systems investigated here, is an essential requirement for a promising solid electrolyte candidate and is used as an approximation of the upper limit of the electrochemical stability.^42^

We now explore the defect chemistry pertinent to the surfaces of the M_3_OX (M = Li or Na; X = Cl or Br) antiperovskites as a route to understand how they may impact the electrochemical performance of these solid electrolytes and determine the ion migration paths that are most energetically favorable. It is well established that vacancy-mediated ion transport is pertinent in antiperovskite solid electrolytes. Indeed, computational studies have revealed metal-halide Schottky defects to be the most energetically favorable types of intrinsic disorder in most antiperovskite systems studied.^22,43−45^ To confirm whether this is also the case for the surfaces of the materials, we first calculated the defect formation energies for isolated metal, halide and oxygen vacancies in the surface slabs to reveal the most energetically favorable defect type in each system. All the defects considered are treated as neutral species.

Defective surface models were created using 3 × 3 × 1 expansions of the metal-halide-terminated (100) surface models, corresponding to large supercells containing 333 atoms (seven antiperovskite layers). Isolated metal, halide and oxygen vacancy defects in these structures were created by systematically removing all nonequivalent atoms in the first three antiperovskite layers of the slabs. Vacancies were also simulated in the bulk materials using 4 × 4 × 4 supercells containing 320 atoms. The defect formation energies (*E*_def_) were calculated using
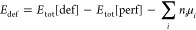
2where *E*_tot_[def] represents the energy of the defective supercell, *E*_tot_[perf] is the energy of the perfect supercell (i.e., with no defects present in the structure), *n*_*i*_ is the number of atoms of type *i* removed (*n*_*i*_ < 0) from the perfect supercell when the defect is created and *μ*_*i*_ is the chemical potential of *i*. This procedure has been successfully used to calculate defect formation energies in a range of antiperovskite systems.^27,28,46,47^

The calculated defect formation energies for each material are displayed in Figures 3(a–c). Given that a lower defect formation energy indicates its more energetically favorable formation, these results reveal key information regarding the defect chemistry in the investigated systems. First, the results reveal that Li and Na vacancies are the most preferable type of vacancy, as compared in Figure 3(d), with the lowest energies consistently observed at the outermost surface layer. This is to be expected given the reduced electrostatic constraints at the surface compared to sublayers and the bulk. If there is a drive for vacancies to segregate strongly to the surface, as suggested by our calculations, this means there will be a region near the surface that has a lower number of vacancies and hence reduced diffusion near the surface and potentially grain boundaries. The energies for halide vacancy formation are consistently lower than those for oxygen vacancies, thereby confirming previous predictions that metal-halide Schottky defects dominate these materials.^22,43,45^ Interestingly, the defect formation energies for the Na-based materials are substantially lower than for the Li-based systems. In contrast, a similar trend was not found when comparing the results for different halides, revealing that the metal species has a greater impact on defect formation at the surfaces and bulk of the investigated antiperovskite systems compared to the choice of the halide.

**Figure 3:**
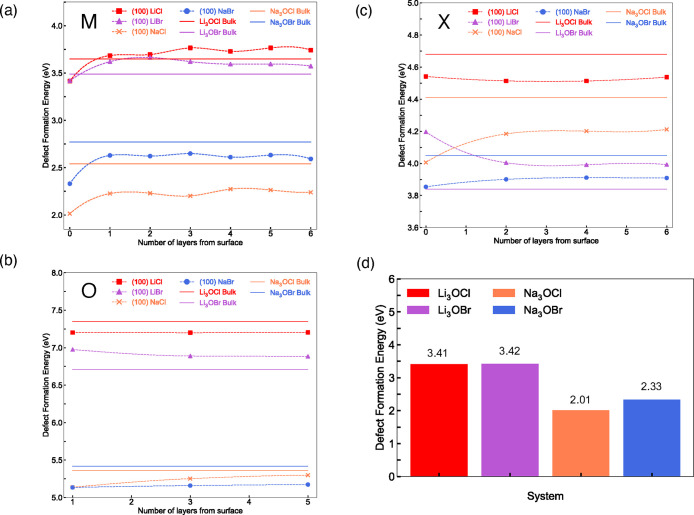
Defect formation energies for (a) M, (b) O, and (c) X vacancies in M_3_OX (M = Li or Na; X = Cl or Br) as a function of surface depth. Bulk values are also presented for comparison. (d) Comparison of defect formation energies for Li and Na vacancies at the first surface layer.

While most of the defect formation energies at the surface converge close to the bulk values, some show a small discrepancy of ∼0.2 eV, which is likely caused by differences in the VBM and CBM between the surface and bulk structures and structural relaxations at the surface that could have an effect deep into the slab. The preference for vacancy formation in the bulk or at the surface appears to be system dependent. In the vast majority of cases, the defects at the outermost surface layer are all lower in formation energy than their bulk equivalents. This can be related to simple electrostatic and geometric factors, where it is easier to remove an ion at the surface due to its reduced/unfavorable coordination and lack of a stabilizing environment. The two exceptions to this rule are oxygen and Br vacancies in bulk Li_3_OBr and at its metal-halide-terminated (100) surface. It is unclear why these particular defects are less favored at this surface but could be related to an unfavorable structural rearrangement or electronic structure.

Considering the prominent role that vacancy-mediated mechanisms play in bulk antiperovskites, herein we explore the migration of Li and Na atoms into vacant sites at the surface layers of the selected systems to systematically assess their contribution to Li- and Na-ion conductivity. We calculated the migration energy barriers for two possible pathways using the nudged elastic band (NEB) method,^48,49^ as illustrated in Figure 4. The first pathway (interoctahedral migration) represents a Li or Na atom at the outermost surface layer migrating to an adjacent vacant site in the same layer. The second pathway (intraoctahedral migration) represents a Li or Na atom in the first subsurface layer migrating to the nearest vacant site at the outermost surface layer. Equivalent pathways were also considered in the bulk materials.

**Figure 4:**
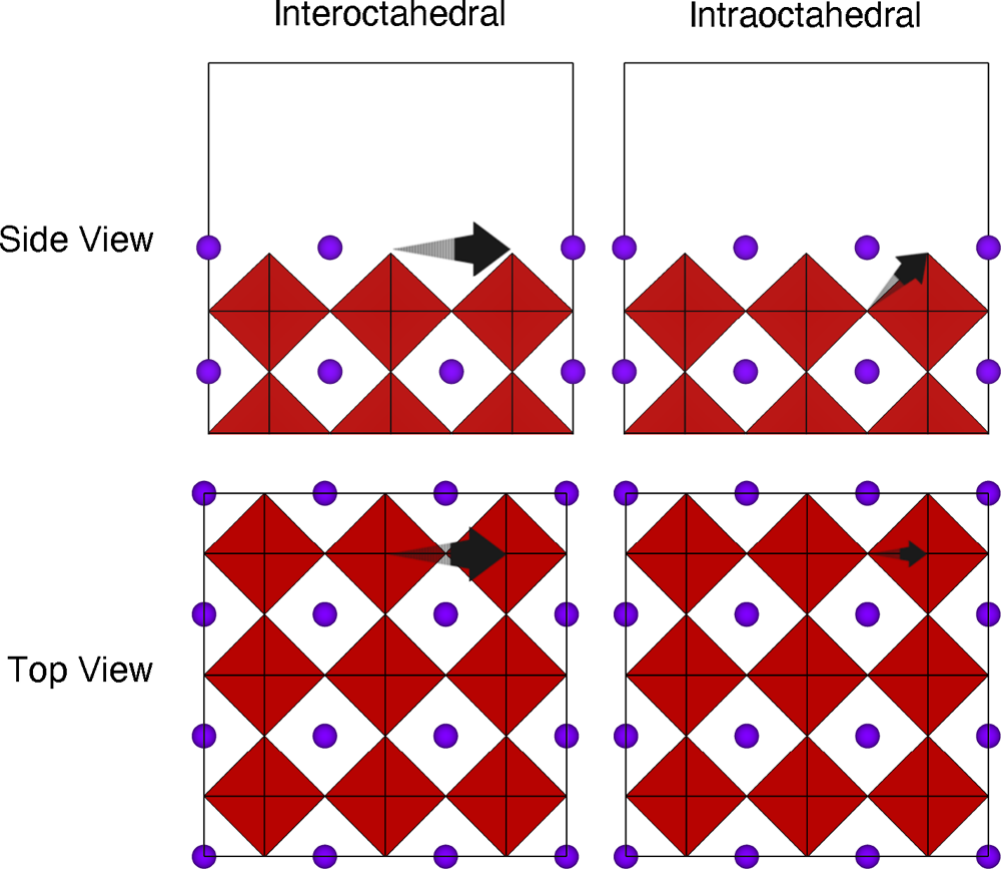
Schematic representations of the two NEB pathways considered for Li and Na migration, i.e., interoctahedral and intraoctahedral hopping, at the metal-halide-terminated (100) surface of M_3_OX (M = Li or Na; X = Cl or Br). The black arrows show the path of the migrating Li or Na atom.

The calculated NEB profiles and energies for Li- and Na-ion migration in bulk M_3_OX (M = Li or Na; X = Cl or Br) and at their metal-halide-terminated (100) surfaces are given in Figures 5(a) and (b), respectively. The migration barriers for both inter- and intraoctahedral hopping in the bulk Li-based systems are in good agreement with previous findings.^50,51^ Similarly, the values obtained for both pathways at the surface of Li_3_OCl agree well with the previous literature report.^27^ The barrier for the interoctahedral pathway at the surface of Li_3_OBr was previously calculated to be 0.58 eV,^28^ which is lower than our value of 0.849 eV.

**Figure 5:**
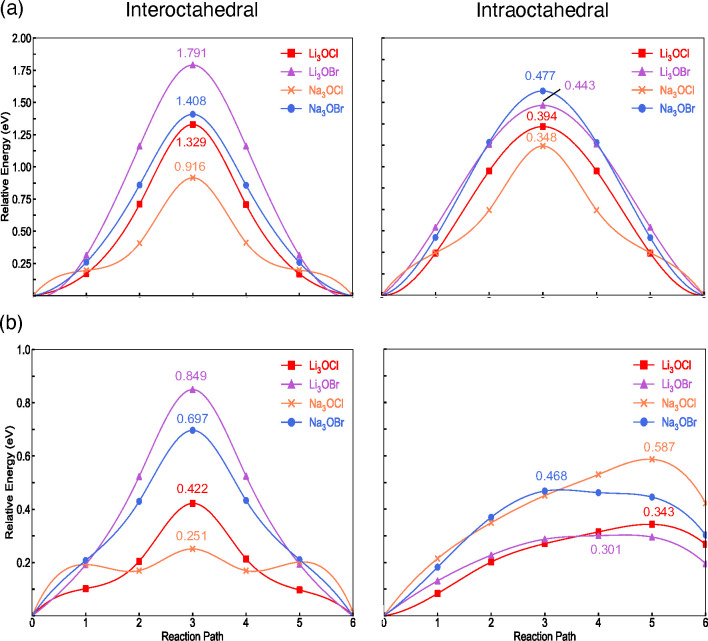
Calculated NEB profiles and energies (eV) for Li- and Na-ion migration in (a) bulk M_3_OX (M = Li or Na; X = Cl or Br) and (b) at their metal-halide-terminated (100) surfaces.

As expected, given the shorter distances involved (∼4 and ∼3 Å for inter- and intraoctahedral migration, respectively), Li- and Na-ion migration via intraoctahedral hopping is more energetically favorable for all bulk systems. The same finding has been confirmed previously for Li_3_OCl^27^ and Li_3_OBr.^28^ The results show that the Cl-based bulk systems display more energetically favorable migrations compared to their Br-based counterparts. While this preference has been reported for Li-based antiperovskites,^51^ this is the first time that it has been shown that it also holds for the Na-based systems. For the bulk systems, the Na-based materials generally show lower barriers than the Li-based systems, with Na-ion transport in Na_3_OCl presenting the lowest energies for both pathways. It is well-known that the exchange of less polarizable atoms for larger, softer and more polarizable atoms (e.g., the replacement of Cl with Br or Li with Na) can weaken the metal-halide interactions, which can potentially improve ionic diffusion.^35,36,52^ Furthermore, ion migration rates are also inherently linked with the distance that the migrating ion must travel and the size of the bottleneck it passes through. Therefore, the enhanced ion transport in the Cl- and Na-based materials likely results from the complex interactions between these interlinked and sometimes competing factors. This phenomenon is also commonly observed in perovskite solid electrolytes.^53^

Analysis of the equivalent surface results also reveals that intraoctahedral migration is typically more energetically favorable. The only exception to this trend is the Na-ion migration that occurs at the surface of Na_3_OCl, which has a remarkably low energy of 0.251 eV. The NEB profile for this migration shows local minima at points 2 and 4 of the pathway that do not appear for the other materials. These minima could indicate potential Na interstitial sites at the surface and a preference for Na Frenkel pair formation at this surface. They could also be related to the presence of the vacuum accelerating transport of the larger Na ion by effectively removing the bottleneck. A reduction in the energy barriers is consistently seen for interoctahedral migration at the surface compared to the bulk. For intraoctahedral migration at the surface, Na_3_OCl now exhibits the highest barrier of 0.587 eV and Li_3_OBr the lowest at 0.301 eV. These results suggest the existence of a complex trade-off between choosing highly polarizable atoms and maximizing bottleneck size for optimized ion migration at both the surfaces and bulk of these materials. Furthermore, it is noteworthy that the barriers (<0.20 eV) for the reverse intraoctahedral hopping process (i.e., from the surface to the first subsurface layer) are significantly lower than from the subsurface layer to the surface. This again illustrates the preference for Li and Na vacancies to exist at the surface. These findings also suggest the potential for Li/Na-deficient grain boundaries with significant interfacial impedance.

Overall, our simulations show that Na_3_OCl is the system that provides the most energetically favorable ionic diffusion across its bulk and at the surface, followed by Li_3_OCl and Li_3_OBr. Considering the importance of effective ion migration in the bulk and at the surface for solid-state batteries, these promising results for Na_3_OCl should encourage future studies of its performance as a solid electrolyte.

In conclusion, we have provided a comprehensive atomistic study of the surfaces of M_3_OX (M = Li or Na; X = Cl or Br) antiperovskites with consideration of their stability, electronic properties, defect chemistry and ion transport. We have shown that the metal-halide-terminated (100) surface is the most thermodynamically stable for all the investigated materials and that Li and Na vacancies closest to the surface are the most favorable defects. We have predicted that the choice of cation has a greater impact on defect formation at the surfaces than the choice of halide. The calculated migration barriers indicate the existence of a trade-off between maximizing atomic polarizability and bottleneck size in the lattice for achieving enhanced ion migration. This study therefore provides important information relating to the thermodynamic and electrochemical stability, defect formation and ion migration rates of antiperovskite solid electrolytes that contributes greatly to the understanding of their application in solid-state batteries.

## Supporting Information Available

Computational methods, including structural details and surface energy, defect formation energy, nudged elastic band and density of states calculations, and the POSCAR file for the optimized (100) LiCl-terminated surface for Li_3_OCl (PDF)
