# Supplementary material for: Influence of Surfaces on Ion Transport and Stability in Antiperovskite Solid Electrolytes at the Atomic Scale
Source: ACS Mater Lett. 2024 Oct 10;6(11):5039–47. doi: 10.1021/acsmaterialslett.4c01777 (PMC11539102; doi:10.1021/acsmaterialslett.4c01777)
Supplement: Supplementary file 1 — tz4c01777_si_001.pdf [file tz4c01777_si_001.pdf]

—Supporting Information—

Influence of Surfaces on Ion Transport and Stability in Anti-Perovskite Solid Electrolytes at the Atomic Scale

Ana C. C. Dutra[a], James A. Quirk[a,b], Ying Zhou[a], and James A. Dawson[a,b]*

[a]*Chemistry − School of Natural and Environmental Sciences, Newcastle University, Newcastle upon Tyne NE1 7RU, UK*

[b]*The Faraday Institution, Didcot OX11 0RA, UK*

*Corresponding Author. E-mail: james.dawson@newcastle.ac.uk

Computational methods

All calculations were performed using the Vienna Ab Initio Simulation Package (VASP)[1] based on density functional theory with the Perdew-Burke-Ernzerhof (PBE)[2] exchange-correlation functional. Interactions between core and valence electrons were described by the projector augmented wave (PAW) method.[3] The valence configurations used in the pseudopotentials were $1s^2\ 2s^1$ for Li, $2p^6\ 3s^1$ for Na, $2s^2\ 2p^4$ for O, $3s^2\ 3p^5$ for Cl and $4s^2\ 4p^5$ for Br.

Structural details

The structural details (e.g., lattice parameters and atomic positions) for all the anti-perovskite systems were obtained from the Materials Project repository.[4] The structural details for Li_2O, LiCl, LiBr, Li metal and the equivalent Na-based materials were required for the purpose of calculating the surfaces energies and were also obtained from the Materials Project repository. The energies of isolated O_2, Cl_2 and Br_2 molecules were determined using cubic cells with a length of 10 Å.

Surface energy calculations

Full geometry optimizations of the bulk systems were carried out by allowing the relaxation of the lattice and atoms. The energy of the electronic ground state was converged to within 10^{-5} eV and the cutoff energy and convergence tolerance were set to 500 eV and 0.01 eV/Å, respectively. Convergence tests with respect to *k*-point sampling density for bulk systems were performed for the optimization of the crystals. A Monkhorst-pack (MP) 4×4×4 *k*-point mesh was found to be adequate for the Li_3OCl, Na_3OCl and Na_3OBr systems, while a MP 5×5×5 *k*-point mesh was needed for Li_3OBr. A MP 3×3×3 *k*-point mesh was used for Na_2O, LiBr and NaBr and a MP 2×2×2 *k*-point mesh was chosen for Li_2O, LiCl and NaCl. For the Li metal and Na metal systems, MP *k*-point meshes of 10×10×10 and 15×15×15 were found to be suitable, respectively.

To create the surface models, we used the periodic "slab" approach, where the geometrically optimized bulk crystal is cut alongside the desired Miller plane (h k l) to expose the target surface. We focused on six-low index symmetric surfaces for each system analyzed, namely, two (100) surfaces, one with a metal-halide termination and one with a metal-oxygen termination, two (110) surfaces, one terminated solely by metal atoms and one terminated by a metal-oxygen-halide ensemble, and two (111) surfaces, one with a metal-halide termination and one terminated solely by oxygens. The symmetric slab models were built with the desired terminations. The metal-halide-terminated (100), metal-oxygen-terminated (100), metal-terminated (110), metal-oxygen-halide-terminated (110), metal-halide-terminated (111) and oxygen-terminated (111) slabs each possessed 52, 53, 47, 43, 44 and 46 atoms, respectively. The dimensions of the slabs are given in Table S1.

Table S1. Dimensions of slab models used for various M_3OX (M = Li or Na; X = Cl or Br) surfaces.

Slab dimensions (Å)				
Termination	Li_3OCl	Li_3OBr	Na_3OCl	Na_3OBr
(100) MX	38.7394	39.5197	45.3855	46.3609
(100) MO	38.8374	39.9697	45.6278	46.7333
(110) M	24.7457	25.2591	29.2400	29.6771
(110) MOX	22.6780	22.9897	27.0494	27.4279
(111) MX	17.9647	18.3858	21.298	21.6682
(111) O	19.5673	19.6744	23.5574	23.7119

Surface energies for all slab models containing a 12 Å vacuum region were calculated by fixing the lattice parameters and allowing ionic relaxations. The specific MP *k*-point mesh used for optimizing each of the surfaces is given in Table S2. The energy of the electronic ground state was converged to within 10^{-5} eV and the cutoff energy and convergence tolerance were set to 500 eV and 0.01 eV/Å, respectively. The effects of the slab size and vacuum thicknesses (12−30 Å) were tested and it was revealed that the surface energy was well converged using the parameters described above.

Table S2. MP *k*-point meshes used for Li_3OCl, Na_3OCl and Na_3OBr surfaces.

Surface	*k*-point mesh
(100) MX	4×4×1
(100) MO	4×4×1
(110) M	3×4×1
(110) MOX	3×4×1
(111) MX	3×3×1
(111) O	3×3×1

Table S3. MP *k*-point meshes used for Li_3OBr surfaces.

Surface	*k*-point mesh
(100) MX	5×5×1
(100) MO	5×5×1
(110) M	4×5×1
(110) MOX	4×5×1
(111) MX	4×4×1
(111) O	4×4×1

Defect formation energy calculations

Defective surface models were created using 3×3×1 expansions of the metal-halide-terminated (001) surface models, corresponding to large supercells containing 333 atoms (seven anti-perovskite layers). Isolated metal, halide and oxygen vacancy defects in these structures were created by systematically removing all non-equivalent atoms in the first three anti-perovskite layers of the slabs. Convergence tests showed that such number of layers were sufficient for all supercells. Vacancies were also simulated in the bulk materials using 4×4×4 supercells containing 320 atoms. Isolated metal, halide and oxygen vacancy defects in these structures were created by systematically removing all non-equivalent atoms in the first three anti-perovskite layers of the slabs. A single *k*-point was used to model these systems. The energy of the electronic ground state was converged to within 10^{-5} eV and the cutoff energy and convergence tolerance were set to 520 eV and 0.01 eV/Å, respectively.

Nudged elastic band calculations

To investigate the ionic migration pathways and energy barriers on the analyzed defective bulk systems and their surfaces, the nudged elastic band (NEB) method[5] implemented in VASP was used. This is a versatile method for finding energy saddle points for ionic migration. NEB calculations were performed by fixing the lattice parameters and allowing ionic relaxations while using a single *k*-point. The energy of the electronic ground state was converged to within 10^{-5} eV and the cutoff energy and convergence tolerance were set to 520 eV and 0.01 eV/Å, respectively. The number of images used between fixed endpoints was five. The defective structures used for such endpoints were optimized using the same electronic ground state convergence and cutoff energy parameters used for the NEB calculations.

Density of states calculations

Density of states (DOS) were calculated for all anti-perovskite systems and surfaces using the hybrid HSE06 functional[6] to improve the bandgap predictions and charge localization. The tetrahedron method with Blöchl corrections was used for the calculation of the DOS plots. The global break condition for the electronic self-consistency-loop set as 10^{-6} eV. The k-point meshes used for the DOS calculations were twice as dense as those used for the same systems during their geometry optimization. The DOS were plotted using the sumo Python toolkit.[7]

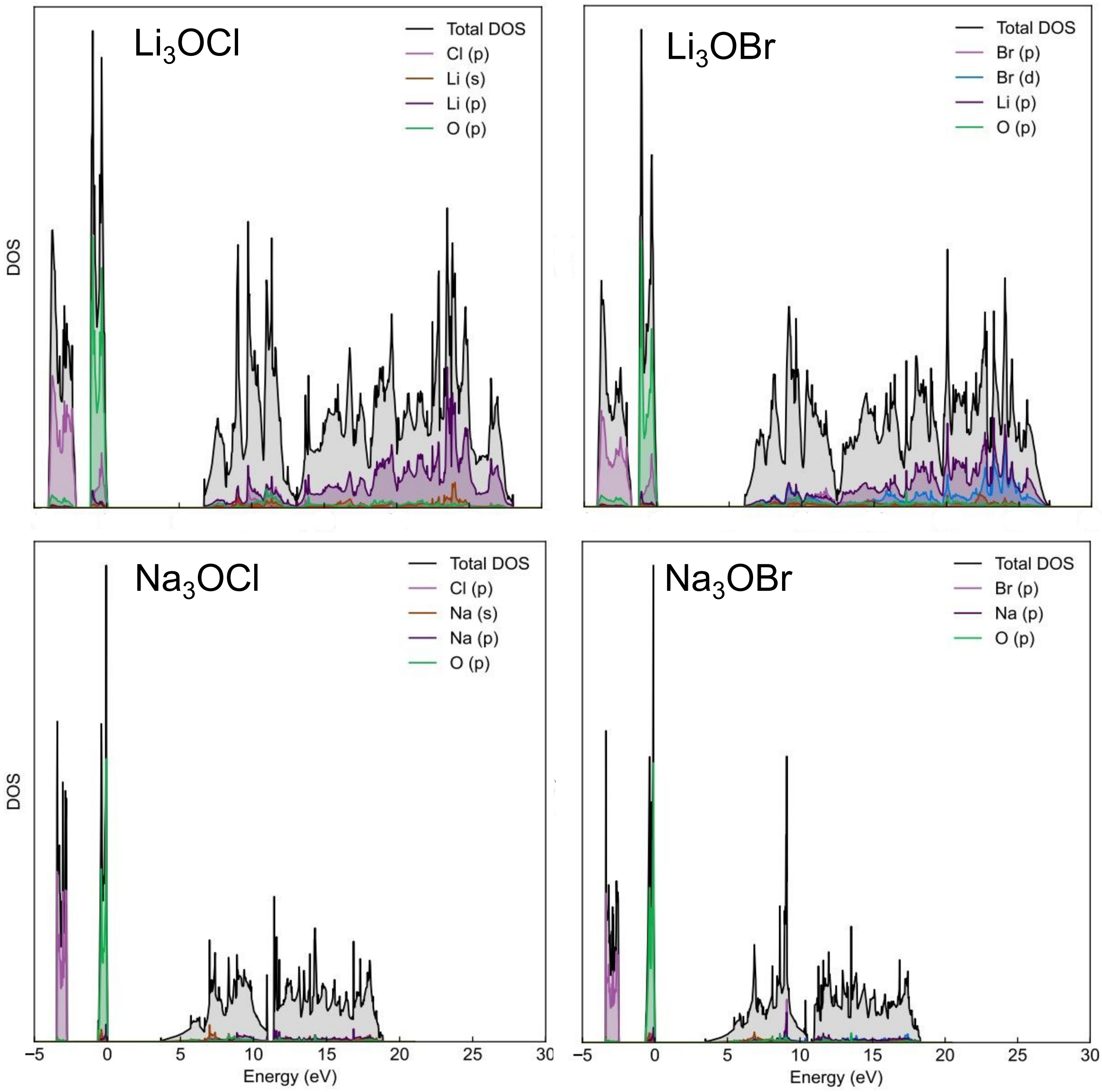

Figure S1. DOS plots for bulk M_3OX (M = Li or Na; X = Cl or Br).

POSCAR file for optimized (100) LiCl-terminated surface for Li_3OCl

```
Li3OCl
1.0
     11.6485252380        0.0000000000        0.0000000000
      0.0000000000       11.6485252380        0.0000000000
      0.0000000000        0.0000000000       50.8284187317
  Cl   Li    O
  99  279   90
Direct
    0.000000000     0.000000000     0.115381040
    0.000000000     0.333333343     0.115381040
    0.000000000     0.666666687     0.115381040
    0.333333343     0.000000000     0.115381040
    0.333333343     0.333333343     0.115381040
    0.333333343     0.666666687     0.115381040
    0.666666687     0.000000000     0.115381040
    0.666666687     0.333333343     0.115381040
    0.666666687     0.666666687     0.115381040
    0.000000000     0.000000000     0.193784550
    0.000000000     0.333333343     0.193784550
    0.000000000     0.666666687     0.193784550
    0.333333343     0.000000000     0.193784550
    0.333333343     0.333333343     0.193784550
    0.333333343     0.666666687     0.193784550
    0.666666687     0.000000000     0.193784550
    0.666666687     0.333333343     0.193784550
    0.666666687     0.666666687     0.193784550
    0.000000000     0.000000000     0.270636380
    0.000000000     0.333333343     0.270636380
    0.000000000     0.666666687     0.270636380
    0.333333343     0.000000000     0.270636380
    0.333333343     0.333333343     0.270636380
    0.333333343     0.666666687     0.270636380
    0.666666687     0.000000000     0.270636380
    0.666666687     0.333333343     0.270636380
    0.666666687     0.666666687     0.270636380
    0.000000000     0.000000000     0.347222567
    0.000000000     0.333333343     0.347222567
    0.000000000     0.666666687     0.347222567
    0.333333343     0.000000000     0.347222567
    0.333333343     0.333333343     0.347222567
    0.333333343     0.666666687     0.347222567
    0.666666687     0.000000000     0.347222567
    0.666666687     0.333333343     0.347222567
    0.666666687     0.666666687     0.347222567
    0.000000000     0.000000000     0.423669219
    0.000000000     0.333333343     0.423669219
    0.000000000     0.666666687     0.423669219
    0.333333343     0.000000000     0.423669219
```

0.333333343	0.333333343	0.423669219
0.333333343	0.666666687	0.423669219
0.666666687	0.000000000	0.423669219
0.666666687	0.333333343	0.423669219
0.666666687	0.666666687	0.423669219
0.000000000	0.000000000	0.500000000
0.000000000	0.333333343	0.500000000
0.000000000	0.666666687	0.500000000
0.333333343	0.000000000	0.500000000
0.333333343	0.333333343	0.500000000
0.333333343	0.666666687	0.500000000
0.666666687	0.000000000	0.500000000
0.666666687	0.333333343	0.500000000
0.666666687	0.666666687	0.500000000
0.000000000	0.000000000	0.576330781
0.000000000	0.333333343	0.576330781
0.000000000	0.666666687	0.576330781
0.333333343	0.000000000	0.576330781
0.333333343	0.333333343	0.576330781
0.333333343	0.666666687	0.576330781
0.666666687	0.000000000	0.576330781
0.666666687	0.333333343	0.576330781
0.666666687	0.666666687	0.576330781
0.000000000	0.000000000	0.652777433
0.000000000	0.333333343	0.652777433
0.000000000	0.666666687	0.652777433
0.333333343	0.000000000	0.652777433
0.333333343	0.333333343	0.652777433
0.333333343	0.666666687	0.652777433
0.666666687	0.000000000	0.652777433
0.666666687	0.333333343	0.652777433
0.666666687	0.666666687	0.652777433
0.000000000	0.000000000	0.729363620
0.000000000	0.333333343	0.729363620
0.000000000	0.666666687	0.729363620
0.333333343	0.000000000	0.729363620
0.333333343	0.333333343	0.729363620
0.333333343	0.666666687	0.729363620
0.666666687	0.000000000	0.729363620
0.666666687	0.333333343	0.729363620
0.666666687	0.666666687	0.729363620
0.000000000	0.000000000	0.806215465
0.000000000	0.333333343	0.806215465
0.000000000	0.666666687	0.806215465
0.333333343	0.000000000	0.806215465
0.333333343	0.333333343	0.806215465
0.333333343	0.666666687	0.806215465
0.666666687	0.000000000	0.806215465
0.666666687	0.333333343	0.806215465
0.666666687	0.666666687	0.806215465

0.000000000	0.000000000	0.884618938
0.000000000	0.333333343	0.884618938
0.000000000	0.666666687	0.884618938
0.333333343	0.000000000	0.884618938
0.333333343	0.333333343	0.884618938
0.333333343	0.666666687	0.884618938
0.666666687	0.000000000	0.884618938
0.666666687	0.333333343	0.884618938
0.666666687	0.666666687	0.884618938
0.166666672	0.166666672	0.118919685
0.166666672	0.500000000	0.118919685
0.166666672	0.833333313	0.118919685
0.500000000	0.166666672	0.118919685
0.500000000	0.500000000	0.118919685
0.500000000	0.833333313	0.118919685
0.833333313	0.166666672	0.118919685
0.833333313	0.500000000	0.118919685
0.833333313	0.833333313	0.118919685
0.000000000	0.166666672	0.155220538
0.000000000	0.500000000	0.155220538
0.000000000	0.833333313	0.155220538
0.333333343	0.166666672	0.155220538
0.333333343	0.500000000	0.155220538
0.333333343	0.833333313	0.155220538
0.666666687	0.166666672	0.155220538
0.666666687	0.500000000	0.155220538
0.666666687	0.833333313	0.155220538
0.166666672	0.000000000	0.155220538
0.166666672	0.333333343	0.155220538
0.166666672	0.666666687	0.155220538
0.500000000	0.000000000	0.155220538
0.500000000	0.333333343	0.155220538
0.500000000	0.666666687	0.155220538
0.833333313	0.000000000	0.155220538
0.833333313	0.333333343	0.155220538
0.833333313	0.666666687	0.155220538
0.166666672	0.166666672	0.193908900
0.166666672	0.500000000	0.193908900
0.166666672	0.833333313	0.193908900
0.500000000	0.166666672	0.193908900
0.500000000	0.500000000	0.193908900
0.500000000	0.833333313	0.193908900
0.833333313	0.166666672	0.193908900
0.833333313	0.500000000	0.193908900
0.833333313	0.833333313	0.193908900
0.000000000	0.166666672	0.232246935
0.000000000	0.500000000	0.232246935
0.000000000	0.833333313	0.232246935
0.333333343	0.166666672	0.232246935
0.333333343	0.500000000	0.232246935

0.333333343	0.833333313	0.232246935
0.666666687	0.166666672	0.232246935
0.666666687	0.500000000	0.232246935
0.666666687	0.833333313	0.232246935
0.166666672	0.000000000	0.232246935
0.166666672	0.333333343	0.232246935
0.166666672	0.666666687	0.232246935
0.500000000	0.000000000	0.232246935
0.500000000	0.333333343	0.232246935
0.500000000	0.666666687	0.232246935
0.833333313	0.000000000	0.232246935
0.833333313	0.333333343	0.232246935
0.833333313	0.666666687	0.232246935
0.166666672	0.166666672	0.270608038
0.166666672	0.500000000	0.270608038
0.166666672	0.833333313	0.270608038
0.500000000	0.166666672	0.270608038
0.500000000	0.500000000	0.270608038
0.500000000	0.833333313	0.270608038
0.833333313	0.166666672	0.270608038
0.833333313	0.500000000	0.270608038
0.833333313	0.833333313	0.270608038
0.000000000	0.166666672	0.308984309
0.000000000	0.500000000	0.308984309
0.000000000	0.833333313	0.308984309
0.333333343	0.166666672	0.308984309
0.333333343	0.500000000	0.308984309
0.333333343	0.833333313	0.308984309
0.666666687	0.166666672	0.308984309
0.666666687	0.500000000	0.308984309
0.666666687	0.833333313	0.308984309
0.166666672	0.000000000	0.308984309
0.166666672	0.333333343	0.308984309
0.166666672	0.666666687	0.308984309
0.500000000	0.000000000	0.308984309
0.500000000	0.333333343	0.308984309
0.500000000	0.666666687	0.308984309
0.833333313	0.000000000	0.308984309
0.833333313	0.333333343	0.308984309
0.833333313	0.666666687	0.308984309
0.166666672	0.166666672	0.347233951
0.166666672	0.500000000	0.347233951
0.166666672	0.833333313	0.347233951
0.500000000	0.166666672	0.347233951
0.500000000	0.500000000	0.347233951
0.500000000	0.833333313	0.347233951
0.833333313	0.166666672	0.347233951
0.833333313	0.500000000	0.347233951
0.833333313	0.833333313	0.347233951
0.000000000	0.166666672	0.385453284

0.000000000	0.500000000	0.385453284
0.000000000	0.833333313	0.385453284
0.333333343	0.166666672	0.385453284
0.333333343	0.500000000	0.385453284
0.333333343	0.833333313	0.385453284
0.666666687	0.166666672	0.385453284
0.666666687	0.500000000	0.385453284
0.666666687	0.833333313	0.385453284
0.166666672	0.000000000	0.385453284
0.166666672	0.333333343	0.385453284
0.166666672	0.666666687	0.385453284
0.500000000	0.000000000	0.385453284
0.500000000	0.333333343	0.385453284
0.500000000	0.666666687	0.385453284
0.833333313	0.000000000	0.385453284
0.833333313	0.333333343	0.385453284
0.833333313	0.666666687	0.385453284
0.166666672	0.166666672	0.423703730
0.166666672	0.500000000	0.423703730
0.166666672	0.833333313	0.423703730
0.500000000	0.166666672	0.423703730
0.500000000	0.500000000	0.423703730
0.500000000	0.833333313	0.423703730
0.833333313	0.166666672	0.423703730
0.833333313	0.500000000	0.423703730
0.833333313	0.833333313	0.423703730
0.000000000	0.166666672	0.461828381
0.000000000	0.500000000	0.461828381
0.000000000	0.833333313	0.461828381
0.333333343	0.166666672	0.461828381
0.333333343	0.500000000	0.461828381
0.333333343	0.833333313	0.461828381
0.666666687	0.166666672	0.461828381
0.666666687	0.500000000	0.461828381
0.666666687	0.833333313	0.461828381
0.166666672	0.000000000	0.461828381
0.166666672	0.333333343	0.461828381
0.166666672	0.666666687	0.461828381
0.500000000	0.000000000	0.461828381
0.500000000	0.333333343	0.461828381
0.500000000	0.666666687	0.461828381
0.833333313	0.000000000	0.461828381
0.833333313	0.333333343	0.461828381
0.833333313	0.666666687	0.461828381
0.166666672	0.166666672	0.500000000
0.166666672	0.500000000	0.500000000
0.166666672	0.833333313	0.500000000
0.500000000	0.166666672	0.500000000
0.500000000	0.500000000	0.500000000
0.500000000	0.833333313	0.500000000

0.833333313	0.166666672	0.500000000
0.833333313	0.500000000	0.500000000
0.833333313	0.833333313	0.500000000
0.000000000	0.166666672	0.538171649
0.000000000	0.500000000	0.538171649
0.000000000	0.833333313	0.538171649
0.333333343	0.166666672	0.538171649
0.333333343	0.500000000	0.538171649
0.333333343	0.833333313	0.538171649
0.666666687	0.166666672	0.538171649
0.666666687	0.500000000	0.538171649
0.666666687	0.833333313	0.538171649
0.166666672	0.000000000	0.538171649
0.166666672	0.333333343	0.538171649
0.166666672	0.666666687	0.538171649
0.500000000	0.000000000	0.538171649
0.500000000	0.333333343	0.538171649
0.500000000	0.666666687	0.538171649
0.833333313	0.000000000	0.538171649
0.833333313	0.333333343	0.538171649
0.833333313	0.666666687	0.538171649
0.166666672	0.166666672	0.576296270
0.166666672	0.500000000	0.576296270
0.166666672	0.833333313	0.576296270
0.500000000	0.166666672	0.576296270
0.500000000	0.500000000	0.576296270
0.500000000	0.833333313	0.576296270
0.833333313	0.166666672	0.576296270
0.833333313	0.500000000	0.576296270
0.833333313	0.833333313	0.576296270
0.000000000	0.166666672	0.614546716
0.000000000	0.500000000	0.614546716
0.000000000	0.833333313	0.614546716
0.333333343	0.166666672	0.614546716
0.333333343	0.500000000	0.614546716
0.333333343	0.833333313	0.614546716
0.666666687	0.166666672	0.614546716
0.666666687	0.500000000	0.614546716
0.666666687	0.833333313	0.614546716
0.166666672	0.000000000	0.614546716
0.166666672	0.333333343	0.614546716
0.166666672	0.666666687	0.614546716
0.500000000	0.000000000	0.614546716
0.500000000	0.333333343	0.614546716
0.500000000	0.666666687	0.614546716
0.833333313	0.000000000	0.614546716
0.833333313	0.333333343	0.614546716
0.833333313	0.666666687	0.614546716
0.166666672	0.166666672	0.652766049
0.166666672	0.500000000	0.652766049

0.166666672	0.833333313	0.652766049
0.500000000	0.166666672	0.652766049
0.500000000	0.500000000	0.652766049
0.500000000	0.833333313	0.652766049
0.833333313	0.166666672	0.652766049
0.833333313	0.500000000	0.652766049
0.833333313	0.833333313	0.652766049
0.000000000	0.166666672	0.691015661
0.000000000	0.500000000	0.691015661
0.000000000	0.833333313	0.691015661
0.333333343	0.166666672	0.691015661
0.333333343	0.500000000	0.691015661
0.333333343	0.833333313	0.691015661
0.666666687	0.166666672	0.691015661
0.666666687	0.500000000	0.691015661
0.666666687	0.833333313	0.691015661
0.166666672	0.000000000	0.691015661
0.166666672	0.333333343	0.691015661
0.166666672	0.666666687	0.691015661
0.500000000	0.000000000	0.691015661
0.500000000	0.333333343	0.691015661
0.500000000	0.666666687	0.691015661
0.833333313	0.000000000	0.691015661
0.833333313	0.333333343	0.691015661
0.833333313	0.666666687	0.691015661
0.166666672	0.166666672	0.729391992
0.166666672	0.500000000	0.729391992
0.166666672	0.833333313	0.729391992
0.500000000	0.166666672	0.729391992
0.500000000	0.500000000	0.729391992
0.500000000	0.833333313	0.729391992
0.833333313	0.166666672	0.729391992
0.833333313	0.500000000	0.729391992
0.833333313	0.833333313	0.729391992
0.000000000	0.166666672	0.767753065
0.000000000	0.500000000	0.767753065
0.000000000	0.833333313	0.767753065
0.333333343	0.166666672	0.767753065
0.333333343	0.500000000	0.767753065
0.333333343	0.833333313	0.767753065
0.666666687	0.166666672	0.767753065
0.666666687	0.500000000	0.767753065
0.666666687	0.833333313	0.767753065
0.166666672	0.000000000	0.767753065
0.166666672	0.333333343	0.767753065
0.166666672	0.666666687	0.767753065
0.500000000	0.000000000	0.767753065
0.500000000	0.333333343	0.767753065
0.500000000	0.666666687	0.767753065
0.833333313	0.000000000	0.767753065

0.833333313	0.333333343	0.767753065
0.833333313	0.666666687	0.767753065
0.166666672	0.166666672	0.806091130
0.166666672	0.500000000	0.806091130
0.166666672	0.833333313	0.806091130
0.500000000	0.166666672	0.806091130
0.500000000	0.500000000	0.806091130
0.500000000	0.833333313	0.806091130
0.833333313	0.166666672	0.806091130
0.833333313	0.500000000	0.806091130
0.833333313	0.833333313	0.806091130
0.000000000	0.166666672	0.844779491
0.000000000	0.500000000	0.844779491
0.000000000	0.833333313	0.844779491
0.333333343	0.166666672	0.844779491
0.333333343	0.500000000	0.844779491
0.333333343	0.833333313	0.844779491
0.666666687	0.166666672	0.844779491
0.666666687	0.500000000	0.844779491
0.666666687	0.833333313	0.844779491
0.166666672	0.000000000	0.844779491
0.166666672	0.333333343	0.844779491
0.166666672	0.666666687	0.844779491
0.500000000	0.000000000	0.844779491
0.500000000	0.333333343	0.844779491
0.500000000	0.666666687	0.844779491
0.833333313	0.000000000	0.844779491
0.833333313	0.333333343	0.844779491
0.833333313	0.666666687	0.844779491
0.166666672	0.166666672	0.881080329
0.166666672	0.500000000	0.881080329
0.166666672	0.833333313	0.881080329
0.500000000	0.166666672	0.881080329
0.500000000	0.500000000	0.881080329
0.500000000	0.833333313	0.881080329
0.833333313	0.166666672	0.881080329
0.833333313	0.500000000	0.881080329
0.833333313	0.833333313	0.881080329
0.166666672	0.166666672	0.155626953
0.166666672	0.500000000	0.155626953
0.166666672	0.833333313	0.155626953
0.500000000	0.166666672	0.155626953
0.500000000	0.500000000	0.155626953
0.500000000	0.833333313	0.155626953
0.833333313	0.166666672	0.155626953
0.833333313	0.500000000	0.155626953
0.833333313	0.833333313	0.155626953
0.166666672	0.166666672	0.232251480
0.166666672	0.500000000	0.232251480
0.166666672	0.833333313	0.232251480

0.500000000	0.166666672	0.232251480
0.500000000	0.500000000	0.232251480
0.500000000	0.833333313	0.232251480
0.833333313	0.166666672	0.232251480
0.833333313	0.500000000	0.232251480
0.833333313	0.833333313	0.232251480
0.166666672	0.166666672	0.308971077
0.166666672	0.500000000	0.308971077
0.166666672	0.833333313	0.308971077
0.500000000	0.166666672	0.308971077
0.500000000	0.500000000	0.308971077
0.500000000	0.833333313	0.308971077
0.833333313	0.166666672	0.308971077
0.833333313	0.500000000	0.308971077
0.833333313	0.833333313	0.308971077
0.166666672	0.166666672	0.385461807
0.166666672	0.500000000	0.385461807
0.166666672	0.833333313	0.385461807
0.500000000	0.166666672	0.385461807
0.500000000	0.500000000	0.385461807
0.500000000	0.833333313	0.385461807
0.833333313	0.166666672	0.385461807
0.833333313	0.500000000	0.385461807
0.833333313	0.833333313	0.385461807
0.166666672	0.166666672	0.461841136
0.166666672	0.500000000	0.461841136
0.166666672	0.833333313	0.461841136
0.500000000	0.166666672	0.461841136
0.500000000	0.500000000	0.461841136
0.500000000	0.833333313	0.461841136
0.833333313	0.166666672	0.461841136
0.833333313	0.500000000	0.461841136
0.833333313	0.833333313	0.461841136
0.166666672	0.166666672	0.538158834
0.166666672	0.500000000	0.538158834
0.166666672	0.833333313	0.538158834
0.500000000	0.166666672	0.538158834
0.500000000	0.500000000	0.538158834
0.500000000	0.833333313	0.538158834
0.833333313	0.166666672	0.538158834
0.833333313	0.500000000	0.538158834
0.833333313	0.833333313	0.538158834
0.166666672	0.166666672	0.614538193
0.166666672	0.500000000	0.614538193
0.166666672	0.833333313	0.614538193
0.500000000	0.166666672	0.614538193
0.500000000	0.500000000	0.614538193
0.500000000	0.833333313	0.614538193
0.833333313	0.166666672	0.614538193
0.833333313	0.500000000	0.614538193

0.833333313	0.833333313	0.614538193
0.166666672	0.166666672	0.691028953
0.166666672	0.500000000	0.691028953
0.166666672	0.833333313	0.691028953
0.500000000	0.166666672	0.691028953
0.500000000	0.500000000	0.691028953
0.500000000	0.833333313	0.691028953
0.833333313	0.166666672	0.691028953
0.833333313	0.500000000	0.691028953
0.833333313	0.833333313	0.691028953
0.166666672	0.166666672	0.767748535
0.166666672	0.500000000	0.767748535
0.166666672	0.833333313	0.767748535
0.500000000	0.166666672	0.767748535
0.500000000	0.500000000	0.767748535
0.500000000	0.833333313	0.767748535
0.833333313	0.166666672	0.767748535
0.833333313	0.500000000	0.767748535
0.833333313	0.833333313	0.767748535
0.166666672	0.166666672	0.844373047
0.166666672	0.500000000	0.844373047
0.166666672	0.833333313	0.844373047
0.500000000	0.166666672	0.844373047
0.500000000	0.500000000	0.844373047
0.500000000	0.833333313	0.844373047
0.833333313	0.166666672	0.844373047
0.833333313	0.500000000	0.844373047
0.833333313	0.833333313	0.844373047

References

(1) Kresse, G.; Furthmüller, J. Efficient Iterative Schemes for Ab Initio Total-Energy Calculations Using a Plane-Wave Basis Set. *Phys Rev B* **1996**, *54* (16), 11169–11186.

(2) Perdew, J. P.; Burke, K.; Ernzerhof, M. Generalized Gradient Approximation Made Simple. *Phys. Rev. Lett.* **1996**, *77*, 3865.

(3) Blöchl, P. E. Projector Augmented-Wave Method. *Phys Rev B* **1994**, *50* (24), 17953–17979.

(4) Jain, A.; Ong, S. P.; Hautier, G.; Chen, W.; Richards, W. D.; Dacek, S.; Cholia, S.; Gunter, D.; Skinner, D.; Ceder, G.; Persson, K. A. Commentary: The Materials Project: A Materials Genome Approach to Accelerating Materials Innovation. *APL Mater* **2013**, *1* (1), 011002.

(5) Henkelman, G.; Uberuaga, B. P.; Jónsson, H. Climbing Image Nudged Elastic Band Method for Finding Saddle Points and Minimum Energy Paths. *Journal of Chemical Physics* **2000**, *113* (22), 9901–9904.

(6) Heyd, J.; Scuseria, G. E.; Ernzerhof, M. Hybrid Functionals Based on a Screened Coulomb Potential. *J Chem Phys* **2003**, *118* (18), 8207–8215.

(7) Ganose, A.; Jackson, A.; Scanlon, D. Sumo: Command-Line Tools for Plotting and Analysis of Periodic Ab Initio Calculations. *J Open Source Softw* **2018**, *3*, 717.
